# Pharmacokinetics and Drug Interactions

**DOI:** 10.3390/pharmaceutics18010067

**Published:** 2026-01-04

**Authors:** Min-Koo Choi, Im-Sook Song

**Affiliations:** 1 College of Pharmacy, Dankook University, Cheon-an 31116, Republic of Korea; minkoochoi@dankook.ac.kr; 2 Research Institute of Pharmaceutical Sciences and College of Pharmacy, Kyungpook National University, Daegu 41566, Republic of Korea

## 1. Introduction

Adverse drug reactions—including those caused by drug–drug interactions (DDIs)—are a major cause of emergency department visits and subsequent hospitalizations in the United States, with studies estimating that 10–30% of these visits are drug-related. Among patients who are hospitalized, 7% experience severe adverse reactions and three out of 1000 die from these reactions [1,2]. Older adults are particularly vulnerable because of polypharmacy, which leads to a disproportionately high number of emergency department visits and hospital admissions related to DDIs [3].

Biomarkers that predict drug response are classified as efficacy or safety indices. Safety biomarkers detect toxicity or adverse effects, while efficacy biomarkers show whether a drug provides the intended therapeutic benefit. Both are closely tied to the activity of drug-metabolizing enzymes and transporters, which determine the concentration and effects of the drug in the body by regulating absorption, tissue distribution, metabolism, and excretion [4,5].

Therefore, drug-metabolizing enzymes and transporters serve as predictive markers for DDIs and herb–drug interactions (HDIs). The US Food and Drug Administration (FDA) recommends evaluating the in vitro inhibitory or inductive effects of new molecular entities on clinically important cytochrome P450 (CYP) isozymes (i.e., CYP1A2, CYP2A6, CYP2B6, CYP2C8, CYP2C9, CYP2C19, CYP2D6, CYP2E1, and CYP3A), UDP-glucuronosyltransferase (UGT) isozymes (i.e., UGT1A1, UGT1A3, UGT1A4, UGT1A6, UGT1A9, and UGT2B7), and drug transporters such as organic cation transporters (OCTs), organic anion transporters (OATs), organic anion transporting polypeptide (OATP), P-glycoprotein (P-gp), and breast cancer-resistant protein (BCRP) during drug development [6]. The correlation between in vitro metabolic and transport properties and clinical PK (pharmacokinetics) during drug development can be demonstrated by statistical results. In the 1990s, over 40% of drug candidates failed clinical trials due to poor PK. After 2000, selecting candidates through an in vitro metabolic and transport screening system reduced attrition rate due to poor PK to below 5% [7,8]. The strong correlation between these in vitro results and clinical outcomes allows drug development to focus on the most promising candidates with reasonable PK properties and the least DDI potential [8,9].

For this purpose, the FDA issued “Guidance for Industries: Drug Interaction Studies,” emphasizing the importance of in vitro and in vivo drug interaction studies on drug-metabolizing enzymes and transporters. The results obtained from this study are included in drug labeling during new drug approval. First released in 2006, the guidelines were updated in 2012 and 2017, with the final version, “Guidance for Industries: In vitro drug interaction studies–Cytochrome P450 enzyme and transporter-mediated drug interactions,” issued in 2020. The European Medicines Agency (EMA), the Ministry of Food and Drug Safety of Korea (MFDS), and Pharmaceuticals and Medical Devices Agency in Japan (PMDA) developed guidelines for DDI studies. These guidelines outline how to assess the DDI potential of a drug candidate to inhibit or induce certain enzymes or transporters using a known probe substrate and to quantify the effect of the interacting drug and predict potential DDIs. In 2024, the International Council for Harmonization of Technical Requirements for Pharmaceuticals for Human Use (ICH) integrated these guidelines into the M12 drug interaction guideline. This guideline addresses pharmacokinetic DDIs, focusing on metabolic enzyme- and transporter-mediated interactions in the development of small molecules. Key changes include standardized in vitro methodologies, defined thresholds for UGT enzyme evaluation, and a less conservative approach to time-dependent inhibition than in previous regional guidance.

Overall, these guidelines highlight the importance of pharmacokinetic DDIs and their possible mechanisms along with probe substrate drugs and commonly used therapeutic drugs. Moreover, HDI mechanisms may also involve the same metabolizing enzymes and transporters-mediated interactions, despite of the diverse chemical structures and modes of action of herbal supplements [6,10]. They also emphasized that pharmacokinetic DDIs and HDIs can be predicted from in vitro inhibition or induction studies on drug-metabolizing enzymes and transporters using combinations of therapeutic drugs with commonly administered drugs or herbal supplements.

## 2. Overview of the Published Articles

A total of 24 papers were published in the Special Issue on “Pharmacokinetics and Drug Interactions,” especially focusing on the HDI potential from the potent inhibitory effect of marker component of herbal medicine. Resveratrol exhibited stereoselective inhibition: trans-resveratrol inhibited CYP1A2, CYP2C19, CYP2E1, and CYP3A time-dependently with K_i_ shift values > 2.0, while cis-resveratrol competitively inhibited CYP1A2. The stereoselective, time-dependent inhibition of resveratrol may result from the formation of a glutathione intermediate with trans-resveratrol [11]. The inhibitory effects of six dibenzocyclooctadiene lignans (schisandrin, gomisin, and wuweizisu C) isolated from *Schisandra chinensis* were evaluated on nine CYP and six UGT isozymes using human liver microsomes. Lignans containing one or two methylene dioxyphenyl groups time-dependently inhibited CYP2B6, CYP2C8, CYP2C9, CYP2C19, and CYP2E1, as revealed using glutathione to trap reactive carbene metabolites [12]. The drug interaction potential arising from the time-dependent inhibition of resveratrol and schisandra lignans should be further assessed using their plasma concentrations, time-dependent kinetic parameters, and the formation of glutathione adducts, along with careful evaluation of potential drug-induced liver injury.

Obtusifolin, an anthraquinone from *Cassia tora* seeds, potently and selectively inhibited CYP1A1 and CYP1A2, with half-maximal inhibitory concentration (IC_50_) values of 0.06 µM and 0.37 µM, respectively. Molecular docking revealed that the strong CYP1A2 inhibition by obtusifolin results from hydrophobic interactions and hydrogen bonding. Since CYP1A1 activates procarcinogens, the drug interaction potential and chemopreventive effects of obtusifolin could be beneficial on chemoprevention with careful consideration on the clinical relevance [13]. In addition to enzyme-mediated drug interactions, transporter-mediated drug interactions were also investigated. Rosmarinic acid is an OAT1 substrate, leading to increased plasma exposure and decreased renal excretion when co-administered with probenecid, a well-known OAT1 inhibitor. Rosmarinic acid inhibited OAT1 and OAT3 transport with IC_50_ values of 60.6 μM and 1.52 μM, respectively, leading to an in vivo pharmacokinetic interaction with furosemide by reducing renal excretion and increasing plasma concentration of rosmarinic acid [14].

After identifying potential single or multiple inhibitions of these metabolizing enzymes and transporters, DDIs between co-administered therapeutic drugs can be quantitatively predicted using physiologically based pharmacokinetic (PBPK) modeling approaches. Because clinical pharmacokinetic data—particularly for complex herbal medicines—are limited, in vivo proof-of-concept studies (animal studies) and Phase I clinical trials are often utilized as alternative approaches [6,15]. Repeated exposure to 1α,25-dihydroxyvitamin D3 significantly increased the renal rOCT1 expression, while decreasing renal rOCT2 and rMATE1, hepatic rOCT1 and N-acetyltransferase 2 (NAT2), and cardiac rOCT3 expression. Intravenous administration of procainamide (10 mg/kg) to 1α,25-dihydroxyvitamin D3-treated rats significantly decreased renal clearance of procainamide and its metabolite, due to reduced rOCT and rMATE1 expression. A PBPK model for the procainamide and its metabolite in rats effectively linked changes in the expressions of rOCTs and rMATE1 transporters to the resulting alterations in the PK of these drugs [16]. Similarly, hepatic OCT1, intestinal OCT3, renal OCT2 on the tubule basolateral membrane, and MATE1/2-K on the tubule apical membrane coordinately regulate metformin disposition. Yang et al. developed a PBPK model of intestinal, hepatic, and renal OCTs and MATEs to predict metformin DDIs. Simulations showed that coadministration of perpetrators increased metformin plasma exposure. Metformin–cimetidine DDI is primarily driven by gastrointestinal transit rate, with additional contribution from transporter inhibition. Among transporters affecting metformin disposition, renal OCT2 and MATEs contributed the most. Therefore, OCT and MATE expression should be monitored in patients taking metformin [17]. However, discrepancies between in vitro and in vivo data can occur. The effects of quercetin on BCRP inducibility and inhibition were observed in vitro. However, in vivo rat BCRP expression remained unchanged after 7 days of quercetin (50–250 mg/kg) administration. In addition, oral administration of quercetin did not alter the PK of sulfasalazine, a probe substrate of BCRP, regardless of dose and duration of quercetin treatment in rats or beagles. Moreover, quercetin metabolites did not inhibit BCRP/mBcrp1, suggesting that quercetin causes negligible in vivo BCRP-mediated drug interactions, likely due to its metabolic inactivation [18].

In addition to investigating DDI potential using probe substrates, evaluating DDI potential between combination drugs is also critical. For example, the DDI potential between voriconazole—a triazole antifungal drug that inhibits CYP3A4 and CYP2C—and tofacitinib, a rheumatoid arthritis medication metabolized by CYP3A4 and CYP2C19, was investigated in rats. The area under the plasma concentration (AUC) of tofacitinib increased by 166% and 171%, respectively, while its time-averaged non-renal clearance decreased by 59.5% compared with tofacitinib alone, indicating its inhibition of tofacitinib metabolism involves the inhibition of Cyp3a2 and Cyp2c11 by voriconazole. The results were consistent with the in vitro results that voriconazole showed noncompetitive inhibition of tofacitinib metabolism in the liver and intestine and the ratio of voriconazole concentration over its IC_50_ value for Cyp3a2 and Cyp2c11 exceeded two [19]. Disease can alter metabolizing enzyme expression levels. In rats with poloxamer 407-induced hyperlipidemia, tofacitinib AUC after intravenous administration was 73.5% higher than in control rats, due to slower nonrenal clearance. The metabolic activity of tofacitinib decreased by 38.6% in hyperlipidemia, due to reduced hepatic Cyp3a2 and Cyp2c11 and intestinal P-gp expression. Similar results were observed in hyperlipidemic rats after oral tofacitinib administration [20]. Amifampridine, approved in 2018 for the treatment of Lambert–Eaton myasthenic syndrome, is mainly metabolized by NAT2. Acetaminophen, a NAT2 inhibitor, strongly suppresses 3-N-acetylamifmapridine formation from amifampridine in rat liver S9 fractions via mixed inhibition. Pretreatment with acetaminophen (100 mg/kg) significantly increased systemic amifampridine exposure and reduced the 3-N-acetylamifampridine to amifampridine AUC ratio, likely via NAT2 inhibition [21].

DDI guidelines recommend using predictive modeling based on in vitro and early clinical data to assess drug safety and efficacy. Depending on mechanistic static or PBPK modeling results, follow-up clinical studies may be required [22]. Rodriguez-Vera et al. developed a PBPK model of phenytoin to assess CYP2C9 and CYP2C19 contributions to 5-(4′-hydroxyphenyl)-5-phenylhydantoin formation and simulate phenytoin exposure after single and multiple intravenous or oral doses of 248–900 mg [23]. Phenytoin acts as a CYP2C9/CYP2C19 substrate and CYP3A4 perpetrator; the PBPK model was used to characterize and predict its DDIs with fluconazole (an inhibitor of CYP3A4, CYP2C9 and 2C19) [24], omeprazole (CYP2C19 substrate/inhibitor and a strong CYP1A2 inducer) [25], and itraconazole (CYP3A4 and P-gp inhibitor) [26]. The simulated and observed DDI AUC ratios (0.89–1.25) support the utility of the PBPK approach in drug development [23]. A similar approach is used for cabozantinib, a tyrosine kinase inhibitor approved for the treatment of advanced renal cell carcinoma, hepatocellular carcinoma, and medullary thyroid cancer. Cabozantinib is primarily metabolized via CYP3A4 and undergoes enterohepatic recirculation [27]. Therefore, Gerner et al. developed a PBPK model including enterohepatic recirculation and validated the model using data from six human clinical studies. Subsequently, the PBPK model was used to simulate DDIs with rifampin and to evaluate the PK profile of subjects with hepatic impairment. The predicted-to-observed ratios for AUC and C_max_ range from 0.9 to 1.2 and 0.8 to 1.1, respectively, suggesting the reliability of the model. The simulated DDI with rifampin leads to a 77% reduction in predicted AUC. Overall, the newly developed PBPK model incorporating enterohepatic recirculation can support the simulation of rifampin interactions and hepatic impairment scenarios [27].

A PBPK model for fexuprazan, a novel potassium-competitive acid blocker, has been developed and validated using integrated in vitro, in vivo, and in silico data [28]. The extent of fexuprazan tissue distribution in humans was estimated using rat tissue-to-plasma partition coefficients and the allometric relationships of distribution volumes across preclinical species. Urinary excretion of fexuprazan was minimal (0.29–2.02%), and the drug was eliminated primarily through hepatic metabolism. The fraction absorbed (0.761), estimated from the PBPK model, was consistent with the physicochemical properties of fexuprazan, including its in vitro solubility and permeability. The predicted oral bioavailability of fexuprazan (38.4–38.6%) was within the range observed in preclinical datasets. The C_max_, AUC, and PK profiles predicted with the PBPK model developed using the learning set were accurately reproduced in the validation sets [28]. Beyond predicting the tissue distribution of fexuprazan, this PBPK model is used to assess DDI potential and to predict intragastric pH under various dosing regimens and co-medication scenarios [29].

Sildenafil, a commonly used therapeutic agent for erectile dysfunction, is primarily metabolized via CYP3A4. Cigarette smoking induces CYP1A2 activity, and both cigarette and cannabis smoking reduce CYP3A4 mRNA in rats or their metabolic activity in in vitro systems [30,31]. To evaluate the effect of cigarettes and/or cannabis smoking on the PK of a CYP3A4-substrate drug, a clinical study was performed. In this study, 36 human participants, including 12 non-smokers, 12 cigarette smokers, and 12 cannabis smokers, were evaluated for the PK, safety, and tolerability of sildenafil following a single 5 mg oral dose. The AUC of sildenafil was significantly higher in cigarette smokers (1156 ± 542 ng·h/mL) and cannabis smokers (967 ± 262 ng·h/mL) than in non-smokers (717 ± 311 ng·h/mL). Collectively, cigarette smoking significantly increases sildenafil exposure without changing pharmacodynamic effects, assessed using the International Index of Erectile Function. The results also suggest that the effects of cigarette and cannabis smoking on CYP3A4 reduce intestinal metabolism of sildenafil, thereby increasing its plasma exposure. The extent of this effect depends on whether CYP3A inhibition occurs primarily in the intestine or the liver [32].

The effects of traxoprodil, a selective NMDA receptor GluN2B subunit antagonist used in the treatment of major depressive disorders, on pituitary and serum hormone levels and the regulation of CYP enzymes in rat liver have been examined in recent studies. Following intraperitoneal administration of traxoprodil (20 mg/kg) for 5 days, the metabolic activity and mRNA expression of CYP1A, CYP2A, CYP2B, CYP2C11, and CYP3A were reduced. After 3 weeks of traxoprodil administration, a decrease in CYP3A enzyme activity and protein levels was maintained. Additionally, a slight decrease in the serum corticosterone concentration was maintained, while growth hormone levels returned to baseline. These findings suggest that the glutamatergic system contributes to the neuroendocrine regulation of CYP enzyme and, therefore, NMDA receptor antagonist may influence metabolic DDIs [33]. The interaction between radiotherapy and the PK of regorafenib has been investigated. In vitro assessments of this interaction were conducted using the human hepatoma Huh-7 cell lines. Regorafenib reduces Huh-7 cell viability in a dose-dependent manner, with apoptosis in Huh-7 cells further enhanced when radiotherapy precedes regorafenib treatment. Radiotherapy reduces the regorafenib AUC by 69–74% in concurrent treatment groups but increases the AUC by 182.8–213.2% in the sequential treatment groups. Both concurrent and sequential radiotherapy significantly decrease the biodistribution of regorafenib in the heart, liver, lungs, spleen, and kidneys, compared to the control (regorafenib × 3 days). Overall, both off-target irradiation and stereotactic body radiation therapy modulate the regorafenib PK and may influence of regorafenib efficacy [34].

The most frequently reported HDIs involve the modulation of drug-metabolizing enzymes and transporters using herbal products, leading to PK changes in substrate drugs. In most cases, PK principles and DDI frame-works used for conventional therapeutic agents are extended to herbal products. This requires accurate quantification of active constituents in herbal preparations, characterization of the PK properties of herbal components, and evaluation of the inhibition constants of each active moiety. In this context, defining PK properties of herbal components provide valuable insights into the appropriate design and interpretation of DDI and PK studies involving herbal supplements. Cannabidiol and its major metabolite, 7-carboxy-cannabidiol, were simultaneously quantified in piglet plasma following intravenous administration of cannabidiol–hydroxypropyl-β-cyclodextrin inclusion complexes. The PK profile of cannabidiol shows a multiexponential decline, characterized by a rapid distribution phase and elimination half-life of 0.25 h and 2 h, respectively. The estimated volume of distribution (3260.35 ± 2286.66 mL) and clearance (1514.5 ± 261.16 mL·h^−1^) indicate that cannabidiol is rapidly distributed to peripheral tissues after administration and is released slowly into the systemic circulation. The concentration–time profile of 7-carboxy-cannabidiol is not consistent with the first-pass metabolism, since 80% of the maximal metabolite concentration is reached at the first sampling time point. Formulation with hydroxypropyl-β-cyclodextrin inclusion complexes may enhance the therapeutic potential of cannabidiol by increasing its intrinsic plasma exposure [35].

Deferasirox is commonly used to manage chronic iron overload in pediatric patients. Galeotti et al. demonstrate that lean body mass (affecting bioavailability and the absorption rate constant), body weight (influencing the volume of distribution), and alanine aminotransferase, aspartate aminotransferase, direct bilirubin, and serum creatinine (influencing hepatic and renal clearance) are key determinants of deferasirox PK. In a cohort of 39 children (26 males) aged 2–17 years who underwent allogeneic hematopoietic stem cell transplantation, significant correlations were observed between deferasirox PK and drug-associated toxicities. Additionally, minimum plasma concentrations of deferasirox >7.0 and 11.5 mg/L were significantly associated with hepatic/renal and hematological toxicities, respectively [36].

Propafenone, a Class 1C antiarrhythmic agent, is primarily metabolized by CYP2D6, CYP1A2, and CYP3A4. Studies show that CYP2D6 polymorphisms influence its PK. To more accurately characterize the effect of CYP2D6 phenotype on the propafenone PK profile, a meta-analysis was performed to integrate all currently available PK studies. Five studies met the inclusion criteria, and analyses were performed to compare PK parameters between poor metabolizers (PMs) and extensive metabolizers (EMs). At the 300 mg dose, the AUC (95% CI), C_max_, and half-life of propafenone in PMs were 15.9 (12.5–19.2) µg·h/mL, 1.10 (0.796–1.40) µg/mL, and 12.8 (11.3–14.3) h, respectively. These values were 2.4-, 11.2-, and 4.7-fold higher than those observed in the EM group and suggested CYP2D6 metabolizer status as a key determinant of propafenone PK. Adjusting the propafenone dose based on CYP2D6 phenotype may help minimize adverse effects and optimize therapeutic efficacy [37].

Oxycodone, a widely used opioid, exhibits substantial inter-individual variability in both its efficacy and PK. In humans, oxycodone is metabolized to oxymorphone, an active metabolite with potent μ-opioid receptor agonist activity. Studies show the critical role of CYP2D6-mediated metabolism of oxycodone to oxymorphone in determining analgesic efficacy. While the recent Clinical Pharmacogenetics Implementation Consortium guidelines do not recommend routine pharmacogenomic testing for oxycodone therapy, pharmacogenetic assessment, particularly CYP2D6 genotyping with attention to potential phenoconversion from concomitant medications, should be considered to optimize oxycodone efficacy and to better characterize the PK profiles of oxycodone and its active metabolite, oxymorphone [38].

Black ginseng extract (BGE), produced from fresh ginseng through nine repeated cycles of steaming and drying, contains higher concentrations of deglycosylated ginsenosides, such as Rg3, Rg5, Rk1, and Rh1, than red ginseng extract (RGE), which undergoes a single steaming cycle. RGE predominantly comprises highly glycosylated ginsenosides, including Rb1, Rb2, Rc, Rd, Re, and Rg1. The safety and tolerability of both extracts were evaluated in a randomized, double-blind, single-dose, crossover clinical trial, and neither extract showed any clinically significant safety concerns. A combination of Rb1, Rg1, and Rg3, marker ginsenosides of RGE, exhibits a 1 h faster absorption rate and 58% higher systemic exposure in RGE than in BGE. Conversely, the combination of Rg3, Rg5, and Rk1, the predominant and most pharmacologically relevant components of BGE, demonstrates an 824% higher absorption in BGE than in RGE. Overall, total ginsenosides show a 79% greater systemic exposure in BGE than in RGE [39]. These findings suggest that ginsenoside plasma exposure correlates with the ginsenoside content of each ginseng product [40].

An interaction study was conducted to evaluate the effects of lactic acid bacteria on the metabolism and PK of ginsenosides in mice. During the in vitro fermentation of RGE with LAB, the levels of protopanaxadiol (PPD) and protopanaxatriol (PPT), final metabolites of ginsenosides not originally present in RGE, significantly increased. Additionally, the concentrations of compound K (CK), Rh1, and Rg3 increased by approximately 30%. Amoxicillin pretreatment significantly decreases both the fecal recovery and plasma concentrations of CK, PPD, and PPT by inhibiting the deglycosylation of ginsenosides following a single oral administration of RGE in mice. Subsequent lactic acid bacteria supplementation for 1 week restores ginsenoside metabolism and plasma ginsenoside levels to those observed in the control. These findings suggest that alterations in the gut microbiota can modulate ginsenoside metabolism and systemic exposure [41], a relationship confirmed in human studies [42].

Recent studies show that energy drink consumption has increased significantly, particularly among adolescents. Caffeine is the primary active compound in these products; however, they contain additional constituents with pharmacological activity. Pain management, cardiovascular health, and hyperlipidemia are among the most common reasons for using herbal supplements [43]. Jahromi et al. reported several categories of HDIs, including cytochrome modulation, additive or synergistic effects, and clinically relevant contraindications [44]. Given that the use of energy drinks and herbal medicines remains largely unregulated and often underreported to healthcare professionals, clinicians must be equipped appropriately to evaluate, integrate, and optimize the use of herbal and other complementary therapies in patient care [43,44].

## 3. Conclusions

DDIs pose a significant challenge in clinical practice, affecting both patient outcomes and therapeutic decision making. For patients, recognizing potential interactions is essential for reducing the risk of adverse drug events, potentially ranging from mild symptoms to severe, life-threatening complications [1,2]. Herbal supplements are not currently integrated into most conventional medical systems. However, as the global use and market share of herbal medicines increases across all age groups, herbal medicines present safety concerns related to DDIs with conventional therapies. In particular, pain management, cardiovascular health, and hyperlipidemia are among the most common reasons for using herbal supplements [43]. Clinical data on DDIs involving herbal products indicate that patients with cardiovascular disease often receive multiple medications, increasing their likelihood of experiencing potential interactions [45]. Therefore, specific herbal products need to be eventually reclassified within conventional medical practice. Healthcare professionals should carefully consider these predictions when selecting alternative therapeutic regimens [2].

In addition, approximately 71% of adverse drug reactions are considered to be at least potentially preventable [2]. Although the frequency of adverse drug reactions attributed to DDIs remains high, accurate prediction of these interactions is essential for safe medication use and the drug-development process. Therefore, evaluating and anticipating DDIs for new drug candidates, alongside commonly used combinations of therapeutic drugs and herbal products, is crucial. As evidence on DDIs accumulates, including the studies presented in this Special Issue, drug interactions could become more preventable or more effectively managed.

## Data Availability

Not applicable.
